# Establishing a Public Resource for Acceptable Surrogate Endpoints to Support FDA Marketing Applications

**DOI:** 10.3389/fmed.2022.820990

**Published:** 2022-02-18

**Authors:** Abena S. Agyeman, Jeffrey N. Siegel, Christopher Leptak

**Affiliations:** Office of New Drugs, Center for Drug Evaluation and Research, Food and Drug Administration, Silver Spring, MD, United States

**Keywords:** surrogate endpoint, 21st Century Cures Act, accelerated approval, traditional approval, biomarker, validated surrogate endpoint, reasonably likely surrogated endpoint

## Abstract

Following a comprehensive and coordinated effort between CBER and CDER, FDA established a table of acceptable surrogate endpoints (SEs) to support drug marketing applications. The publicly accessible SE Table was first published in 2018 as a response to the 21st Century Cures Act legislation and is updated every 6 months to reflect current FDA thinking. The criteria for the table headings and content were chosen to foster succinctness and consistency, while reflecting the degree of scientific understanding for each listed SE. Prior to the publication of the SE table there was the misconception that FDA only approved drugs based on a limited number of SEs. Contrary to this viewpoint, the SE table demonstrates that FDA frequently uses SEs as they are used in over 100 disease/use and patient population combinations. This article describes the considerations and approach taken when establishing the SE table as well as a discussion of the benefits and limitations of the SE table when used by various stakeholders.

## Introduction

To expedite patients' access to new interventions for serious conditions, FDA has a long history of openness toward the appropriate use of surrogate endpoints (SE) as part of drug^1^ development and approval (1, 2). SEs are biomarkers used in clinical trials that predict clinical benefit but are not themselves a direct measure of that clinical benefit. Common examples include blood pressure, hemoglobin A1c, and HIV viral load. SEs may allow clinical trials to be conducted faster, at a lower cost, and/or for shorter durations.

From a U.S regulatory perspective, a SE is characterized as validated, reasonably likely, or candidate based on the level of clinical validation and whether sufficient evidence exists to demonstrate the prediction of the specified clinical benefit. Validated SEs have been shown to predict clinical benefit based on clear causal/mechanistic rationale of the disease process and correlative clinical data with respect to clinical benefit; validated SEs can be used to support traditional approval (3). Reasonably likely SEs are supported by strong mechanistic data and/or epidemiologic rationale, but the available clinical data do not yet clearly demonstrate that they predict clinical benefit. These SEs can be used to support accelerated approval of a product that provides a meaningful therapeutic benefit over existing treatments for a serious or life-threatening disease or condition. Post-marketing confirmatory trials have been required to verify and describe the anticipated effect of the SE on irreversible morbidity or mortality or other clinical benefit (4)^2^. A candidate SE is still under evaluation for its ability to predict clinical benefit (3) and may not be relied upon to support approval of a marketing application.

Section 3011 of the 21st Century Cures Act (Cures) established section 507 of the Federal Food, Drug, and Cosmetic Act (FD&C Act). Section 507 (e) includes a requirement that the FDA publish “a comprehensive list of…(ii) all surrogate endpoints which were the basis of approval or licensure (as applicable) of a drug or biological product” under both accelerated and traditional approval provisions. There was also a requirement to update the table every 6 months.

Prior to Cures, there was a long-standing internal and external interest to have an FDA-cleared list of surrogate endpoints (SEs) that could be used as the primary basis of product approval. FDA recognized this need and made multiple attempts to consolidate this information into a common resource. However, the lack of consensus definitions, limitations of internal knowledge management systems, and differing approaches to the display of SE-related information impeded the goal. The alignment of the passage of the Cures legislation and the development of consensus definitions of terms relevant to SEs, addressed prior challenges. The taxonomy of consensus definitions is known as the Biomarker Endpoints and other tools (BEST) glossary (3) established by the FDA-NIH Biomarker Working Group in 2015.

The SE Table is a valuable resource for not only FDA but also multiple external stakeholders, including but not limited to: pharmaceutical and biotech companies, academia, consortia, other government agencies, other regulatory bodies, private insurance, and special interest groups such as patient and pharmaceutical advocacy associations. Since its first publication in July 2018, the SE table has undergone periodic updates, the most recent being the sixth. A new FDA SE webpage (5) was simultaneously launched that links to the SE table (6) and Considerations for Discussion of a New Surrogate Endpoint at the PDUFA VI Type C meetings (7).

This manuscript describes the process FDA used to establish the public-facing SE table that was ultimately cleared by the Center for Drug Evaluation and Research (CDER) and Center for Biologics Evaluation and Research (CBER). It describes the rationale behind what data are displayed and how they are organized to be as consistent, clear, and concise as possible. Lastly, it describes how the table can be used by stakeholders.

## Criteria for the Surrogate Endpoint Table

Since specific requirements for the SE table were not detailed in the Cures legislation, we consolidated information from previous SE collation efforts and curated the information against BEST glossary definitions and the SE definitions put forward in Section 507 of the FD&C Act^3^.

This effort was specifically focused on biomarkers used as SEs to support either full or accelerated drug approval. In addition to prior SE metadata research efforts, we also gathered information from other internal knowledge management sources, existing disease-specific guidances, and drug labeling. To make the SE table as comprehensive and useful as possible, we did an extensive collaborative outreach to the FDA clinical divisions to gather information about regulatory use of SEs, curated the SE information, and discussed SEs that could support drug approval even though they had not yet been used by a specific drug program. The last additional step was taken so that the table could be more informative and allow for increased transparency.

Since the legislation called for a comprehensive list of SEs with no defined start date, it was decided that the table would include SEs from as far back as the FDA data bases and institutional knowledge could support. While some SEs may be appropriately used in both adult and pediatric populations for the same disease, the adult, and pediatric SE tables were intentionally kept separate to emphasize which ones had been explicitly studied in pediatric patients.

## Data Sources

Data sources that are used to curate the SE table were as follows:

- Previous FDA efforts to compile a list of SEs.
- Internal metadata repositories.
- End of Phase II and Special Protocol Assessment meeting minutes where SEs were discussed. (1997 to present)
- Divisional knowledge especially for historical SEs for which there have not been recent drug approvals but would still be acceptable, e.g., thyroid-stimulating hormone for patients with hypothyroidism.
- Drug labels (8, 9).
- FDA drug-specific guidances (10).
- FDA Listing of Established Pharmacologic Class Text Phrases (11).

## SE Table Column Headings

An important consideration was how best to display the data to make it as consistent as possible across divisions while also providing sufficient context for external stakeholders. To make the SE table informative and useful, we decided to generalize the information when appropriate and supported by the science as an over-arching principle (e.g., instead of listing all the possible oncologic tumor types, the general term solid tumor was used). The final column headings are as follows; disease/use, patient population, surrogate endpoint, type of approval appropriate for (accelerated or traditional), and drug mechanism of action.

Instead of including a column for indications, we include disease/use and patient population columns because:

1. SEs are not always used in the context of a disease (e.g., benign hematology, vaccinations, or antiseptics)
2. SEs are not always indication-specific (e.g., hemoglobin could be an SE for a variety of hematologic conditions/indications)
3. Verbatim listing of approved indications may be too long to put in a table
4. Labeling changes over time would not allow for consistency and ease of updating the table every 6 months.

Since the 21st Century Cures Act required the listing of SEs approved using both the accelerated and traditional pathways, the type of approval is a heading for the SE table. When multiple disease/use and patient populations are associated with a SE, both types of approval are listed. If over time a reasonably likely SE becomes a validated SE this will be reflected on the table by changing from accelerated to traditional approval during the update. Likewise, if the scientific understanding of a disease changes such that the use of the SE is no longer appropriate, the SE table will be updated to reflect this change.

Drug names were not included on the table due to proprietary disclosure issues with respect to drugs still in development, especially due to the listing of SEs that would be acceptable as a primary efficacy endpoint even though they have not yet been used for an approved product. In addition, listing individual drug names would make the table difficult to update every 6 months. Instead of specific drug names, the drug mechanism of action is a column heading because it avoided these issues and put into context the relationship of the SE with the pathophysiological pathway of the disease that the drug targets. The mechanism of action is described based on the FDA Established Pharmacologic Class database (11).

## Descriptions in SE Table

Overall, the criteria for the descriptions in the table are to be as brief and general as possible based upon the scientific support and as accepted by the FDA clinical review division. These criteria allow for consistency and makes the SE table easier to update. An example of how the descriptions were adapted from various data sources and finalized on the SE table can be found for pulmonary tuberculosis (See Table 1).

**Table 1:** An example of how the descriptions for the SE table were adapted from various data sources [drug label for SIRTURO® (bedaquiline) (8) and the guidance (12)] can be found for pulmonary tuberculosis.

**Data source**	**Disease or use**	**Patient population**	**Surrogate endpoint**	**Type of approval appropriate for**	**Drug mechanism of action**
Labeling	Pulmonary multi-drug resistant tuberculosis	Part of combination therapy in the treatment of adult and pediatric patients (5 years and older and weighing at least 15 kg) with pulmonary multi-drug resistant tuberculosis (MDR-TB)	Time to sputum culture conversion to negative defined as the interval in days between the first dose of study drug and the date of the first of two consecutive negative sputum cultures collected at least 25 days apart during treatment	Accelerated	Diarylquinoline antimycobacterial
Guidance	Pulmonary tuberculosis	Patients with pulmonary tuberculosis	Rate of sputum culture conversion from positive to no growth of M. tuberculosis during treatment, either as a time to conversion analysis or at a fixed time point (e.g., at 2 months)	Accelerated	
Final adult SE table	Pulmonary tuberculosis	Patients with active pulmonary tuberculosis	Sputum culture conversion to negative	Accelerated	Antimicrobial

To streamline the SE table, descriptors for measurements such as time points and amounts^4^, rates^5^, and metric units^6^ are excluded when possible. Details of concomitant or prior therapies, and type of tumor mutations are also generally omitted in the patient population description because such information can be easily found in drug labels of interest. Although critical to the use of the SE, the measurement method used isn't included in the table since platforms evolve over time. Including this information would make it difficult to update and therefore keep accurate over time. Considerations to keep in mind when developing a measurement method for an SE can be found elsewhere (7). Information that was deemed important for stakeholders wanting to use SEs in their drug development programs were the age range for pediatrics (which are listed in a separate column in the pediatric table) and the biological matrix that the SE is measured in (e.g., sputum, blood, plasma, skeletal muscle etc.).

There are differing degrees to the generalization of the disease/use and patient population columns on the SE table. Due to the ever-changing oncology landscape, with many approvals for specific indications occurring regularly, there are many different SEs and specific patient population combinations. As such, oncology disease descriptions are generalized (e.g., solid tumors, hematological malignancies, and benign hematology) based on the dynamic nature of this disease area. The patient populations are generalized to the organ site or system (e.g., breast cancer, prostate cancer, acute myeloid leukemia etc.). If more details were to be included the table would be significantly longer and difficult to update. SEs with fewer disease/use and patient population combinations are individually listed (e.g., bacterial count, serum uric acid, and plasma phenylalanine) and contain more detail.

The level of detail of the drug mechanism of action differs based on if there is a common mechanism vs. many mechanisms. For example, “antiviral” is a descriptor used because it encompasses a common mechanism for a number of drugs for diseases such as Hepatitis B, C, D, HIV-1, or CMV. If a SE is on a pathophysiological pathway that can be targeted by many drugs with different mechanisms of action, the term “mechanism agnostic” is used. This is the case for SEs such as proteinuria, pathological complete response, and forced vital capacity. For SEs that apply to drug mechanisms of action that are not too many to list, the mechanisms of action are listed individually (e.g., serum phosphate and urine free cortisol).

## Discussion

Validated surrogate endpoints (SE's) are endpoints supported by a clear mechanistic rationale and clinical data providing strong evidence to predict a specific clinical benefit for use as a primary efficacy endpoint in a pivotal clinical trial (13). A reasonably likely SE is supported by strong mechanistic and/or epidemiologic rationale such that an effect on the surrogate endpoint has a predictive potential for a clinical benefit but post-approval confirmatory studies would be needed to establish that a drug approved based on an effect on this SE confers a clinical benefit (14). It is important to note that concerns have been raised about the use of surrogate endpoints for approval of new therapies (15, 16). However, the use of SEs in drug development programs is context-dependent and therefore the reasoning behind how or why a particular SE was used as either a reasonably likely or validated SE is beyond the scope of this article, however a more detailed explanation of reasonably likely and validated SEs can be found in the BEST resource chapters on these topics (17, 18).

The surrogate end point table includes both validated and reasonably likely SE's as mandated by Cures. The table includes SE's used for approvals in adult populations and a separate table for pediatric SE's.

There has been an effort to maintain consistency in descriptions throughout the SE table; however, this is balanced with an effort to make the descriptions succinct and informative. This has inevitably led to differences in level of detail captured for different disease areas, as described above. Of note, the table includes both SEs that have been used in drug approvals and SEs that have not yet been used in drug approvals but where FDA has indicated acceptance of these SEs in guidances or other documents. As such the table should be used as a reference point that stakeholders can use initially followed by further research and/or contact with the FDA to discuss the use of a SE in their drug development programs.

It is also evident from the SE table that FDA has accepted surrogates for approval of therapies across numerous therapeutic areas including highly prevalent conditions (e.g., HbA1c, LDL-cholesterol, FEV1, and blood pressure) and rare diseases (e.g., urine free cortisol, reduction of GL-3 inclusions).

Since it was published in July 2018 the SE table has been beneficial both internally to FDA and to external stakeholders. The SE table serves as a reference point that allows these stakeholders to quickly determine if a SE has already been used in a specific context and can help to identify areas where further SE development is needed. Internally, developing and updating the SE table has been a very valuable exercise for FDA. It allows broad cross-divisional discussions about how SEs are being used, assessing whether the approach to using them is similar or different across the Agency and thereby allowing SE-related policy to be implemented. The SE table is also helpful for biomarker qualification, which is the approval of biomarkers across drug development programs, because it provides a comprehensive reference of the use of SEs across disease areas and patient populations.

A prime example of how the SE table is used by both FDA and external stakeholders is for PDUFA VI Type C SE meetings. These are special meetings for SEs that are novel for a specific context of use. The SE table allows both parties to be on the same page in terms of which SEs have and have not already been approved as a primary efficacy endpoint in a particular disease area for a given patient population.

## Data Availability Statement

The original contributions presented in the study are included in the article/supplementary material, further inquiries can be directed to the corresponding author/s.

## Author Contributions

AA did the research and wrote the paper. JS edited the manuscript. CL supervised AA in research and edited the manuscript. All authors contributed to the article and approved the submitted version.

## Conflict of Interest

The authors declare that the research was conducted in the absence of any commercial or financial relationships that could be construed as a potential conflict of interest.

## Publisher's Note

All claims expressed in this article are solely those of the authors and do not necessarily represent those of their affiliated organizations, or those of the publisher, the editors and the reviewers. Any product that may be evaluated in this article, or claim that may be made by its manufacturer, is not guaranteed or endorsed by the publisher.

## Footnotes

^1^For the purposes of this guidance, all references to *drugs* or *drug products* include both human drugs and biological drug products regulated by CDER and CBER unless otherwise specified.

^2^Section 506(c)(3)(A) of the FD&C Act and §§ 314.510 and 601.41.

^3^Section 507(e) FD&C: “The term surrogate endpoint' means a marker, such as a laboratory measurement, radiographic image, physical sign, or other measure, that is not itself a direct measurement of clinical benefit, and—“(A) is known to predict clinical benefit and could be used to support traditional approval of a drug or biological product; or “(B) is reasonably likely to predict clinical benefit and could be used to support the accelerated approval of a drug or biological product in accordance with section 506(c)”.

^4^E.g: “Attainment of plasma methotrexate concentration ≤1 μmol/L at 15 minutes that was sustained for up to 8 days following the initial injection” [drug label] for VORAXAZE® (glucarpidase) (7) is described on the SE table as “patients with delayed methotrexate clearance due to impaired renal function”.

^5^E.g: estimated glomerular filtration rate = ml per minute per 1.73 m^2^ of body-surface area.

^6^E.g: mm for probing pocket depth.
